# Genetic and epigenetic factors shape phenotypes and outcomes in systemic lupus erythematosus – focus on juvenile-onset systemic lupus erythematosus

**DOI:** 10.1097/BOR.0000000000001072

**Published:** 2024-12-11

**Authors:** Amandine Charras, Linda T. Hiraki, Laura Lewandowski, Christian M. Hedrich

**Affiliations:** a Department of Women's and Children's Health, Institute of Life Course and Medical Sciences, University of Liverpool, Liverpool, UK; b Genetics & Genome Biology, Research Institute, and Division of Rheumatology, The Hospital for Sick Children, & Division of Rheumatology, The Hospital for Sick Children, Toronto, Ontario, Canada; c National Institute of Arthritis and Musculoskeletal and Skin diseases, NIH, Bethesda, Maryland, USA; d Department of Rheumatology, Alder Hey Children's NHS Foundation Trust, Liverpool, UK

**Keywords:** childhood, epigenetic, genetic, juvenile, systemic lupus erythematosus

## Abstract

**Purpose of review:**

Systemic lupus erythematosus (SLE) is a severe autoimmune/inflammatory disease. Patients with juvenile disease-onset and those of non-European ancestry are most severely affected. While the exact pathophysiology remains unknown, common and rare gene variants in the context of environmental exposure and epigenetic alterations are involved. This manuscript summarizes the current understanding of genetic and epigenetic contributors to SLE risk, manifestations and outcomes.

**Recent findings:**

Though SLE is a mechanistically complex disease, we are beginning to understand the impact of rare and common gene variants on disease expression and associated outcomes. Recent *trans*-ancestral and multigenerational studies suggest that differential genetic and environmental impacts shape phenotypic variability between age-groups and ancestries. High genetic burden associates with young age at disease-onset, organ involvement, and severity. Additional epigenetic impact contributes to disease-onset and severity, including SLE-phenotypes caused by rare single gene variants. Studies aiming to identify predictors of organ involvement and disease outcomes promise future patient stratification towards individualized treatment and care.

**Summary:**

An improved understanding of genetic variation and epigenetic marks explain phenotypic differences between age-groups and ancestries, promising their future exploitation for diagnostic, prognostic and therapeutic considerations.

## BACKGROUND

Systemic lupus erythematosus (SLE) is a chronic autoimmune/inflammatory disease with significant morbidity and mortality [1,2]. Severity and disease outcomes vary between ancestries and age-groups with non-European and pediatric patients being most severely affected [3–6]. Although the exact pathophysiology of SLE remains unclear, the importance of genetic factors has been established [3,7,8] with increased risk of SLE in twins and siblings of those with SLE [9,10]. While genetic factors increase the risk for SLE significant genetic variability and environmentally mediated factors may explain phenotypic differences between patients, ancestries and age-groups [11].

Differences in disease presentation and progression between adult- (aSLE) and juvenile-onset (j)SLE patients may be caused by variable composition of inherited and acquired factors. Notably, compared to aSLE, an increased prevalence of common and rare genetic variants has been reported in jSLE, and increased genetic burden may result in early disease expression and more severe phenotypes [11–13,14^▪^]. However, even within this relatively small group of patients with monogenic risk variants, phenotypic variability exists. Thus, additional genetic and acquired (epigenetic) factors emerged as disease-modifying factors that may be used as predictive markers [15^▪^,^16^]. 

**Box 1:**
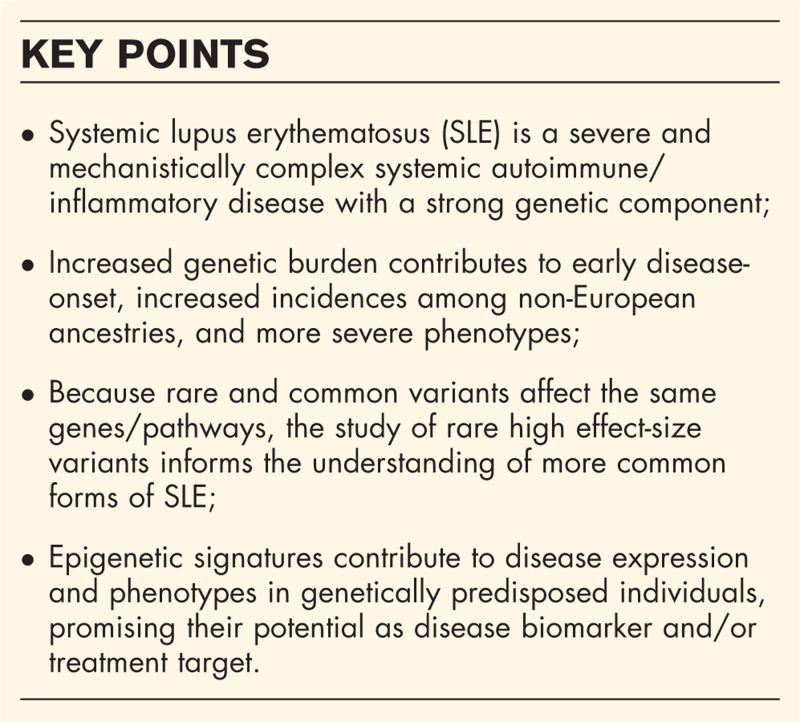
no caption available

## COMMON VARIANTS

For most people with SLE, it is a complex polygenic disease. Hundreds of SLE-associated single nucleotide polymorphisms (SNPs) have been identified from genome wide association studies (GWAS) primarily including adult participants [17–19]. While individual SNPs and extended haplotypes increase an individual's risk for SLE, their impact on gene function does not sufficiently explain disease expression. As a result, SNPs only explain a fraction of the heritability of SLE [20–22]. Because of the relatively low impact of SLE-associated SNPs on gene function, polygenic risk scores (PRSs) have been proposed to estimate risk [23] and account for 30–73% of SLE heritability [24,25^▪▪^]. Missing heritability is due to several factors, including: (a) Limitations in estimating epigenetic changes, gene:gene and gene:environment interactions; (b) Missed ancestry-specific alleles through relative under-representation of non-European ancestries; (c) rare SLE-associated variants are not captured by GWAS, but may be more prevalent among jSLE when compared to aSLE patients [1,2,11,20,21,24,25^▪▪^].

While GWAS have improved our understanding of SLE genetics, available studies have limitations. Population stratification, or confounding by genetic ancestry, was frequently addressed by restriction to European participants [26]. This is a particular challenge when aiming to understand a disease that is more prevalent and severe among non-European populations [6,27]. In recent years, *meta-*GWAS in stratified ancestral populations [18,28,29,30^▪▪^] and *trans-*ancestral GWAS [17,19,25^▪▪^,^30^^▪▪^,^31^] demonstrated shared and distinct SLE risk loci across global populations. A *trans-*ancestral *meta-*GWAS of European-American (Cases/controls: 6748/11 516), African-American (2970/2452) and Hispanic-American (1872/2016) SLE patients identified 22 shared risk loci, which included 11 previously unidentified regions [17]. *Meta-*GWAS of East-Asian (6707/16 047) and European (28 662/43 408) populations identified 79 SLE risk loci shared between populations, with 9 loci showing ancestry differential effects [25^▪▪^]. Continued expansion of historically underrepresented global populations will result in the identification of currently unknown SLE-associated variants.

The human leukocyte antigen (HLA) system on chromosome 6 is the most polymorphic region of the genome [32] and has strong associations with immune-mediated diseases. It was the first genetic region associated with SLE [33–35] and demonstrates some of the strongest associations with SLE risk [17–19,25^▪▪^]. A study across 11 autoimmune/inflammatory phenotypes, accessing whole exome sequencing (WES) data from UK biobank participants, identified more HLA risk alleles in SLE when compared to other immune-mediated diseases [36^▪▪^]. However, there are likely still HLA alleles and extended haplotypes to be discovered.

Although most GWAS studies were performed in aSLE cohorts, risk alleles identified are also relevant in jSLE. Several studies have reported an inverse association between the number of risk alleles and age at diagnosis [12,17,37^▪▪^,^38^]. Because risk alleles and PRS burden associate with disease severity, increased genetic burden in children may also contribute to more severe phenotypes [4,12,37^▪▪^,^38^]. A recent study testing associations between SLE risk alleles and age at diagnosis identified and intronic region of *CCDC113* to be associated with young age at diagnosis [39]. The study also completed the first GWAS of jSLE (Cases/controls: European: 199/3671; East-Asian: 556/409) identifying two SLE-associated regions on chromosome 6 mapping to a *TSBP1-AS1* intron and *HLA-DQA1* (previously associated with aSLE).

Another limitation of GWAS relates to the prediction of impact of SNPs and/or extended haplotypes on gene function. Recently, innovative analytical models have been applied to datasets, identifying previously not appreciated SLE-associated variants [40]. A multiancestral study of SLE GWAS cohorts, including participants of East-Asian (cases/controls: 5877/188 558), European (14 355/505 956) and Admixed-American (1393/2327) ancestries, performed multitrait analyses using summary statistics from 13 other autoimmune/inflammatory diseases [40]. Authors confirmed 79 and identified 27 “novel” SLE risk loci. Lastly, transcriptome-wide analyses and cell type enrichment using gene expression prediction identified an additional 22 risk loci. Developments are not limited to the association of genetic variability with disease risk; pathway prediction and computational drug repurposing may also predict future therapeutic interventions [40].

A central aim of genetic studies is associating genetic variability with clinical manifestations, complications, and disease outcomes. Across studies, detailed clinical information is frequently lacking, limiting the ability to predict genotype:phenotype associations. A recent North American study involving children and adults with SLE observed an association between 39 non-HLA SNPs [odds ratio (OR) = 1.26; 95% confidence interval (CI):1.09–1.46; *P* = 0.0006] and lupus nephritis (LN), which was strongest among children of European ancestry when compared to aSLE patients or children of non-European ancestry [41^▪^]. A Taiwanese study observed associations between a combination of 27 SLE-associated SNPs and LN onset at younger ages [38]. A study in 319 jSLE patients from the UK identified 10 type I interferon (IFN)-related jSLE quantitative trait loci to be associated with young age at disease-onset (*IRAK1*, *TLR7*, *IFIH1*, *STAT4*, *TYK2*, *IRF8*), global disease activity (*IFIH1*, *STAT4*, *IRF8*), and/or mucocutaneous involvement (*TLR7*, *IFIH1*) [42] (Table 1). Studies linking SLE-associated osteonecrosis with SLE risk alleles yielded variable results. A GWAS involving 636 SLE patients with steroid-associated osteonecrosis (95 588 controls, Japanese Biobank) identified previously not know risk loci, namely *MIR4293/MIR1265* (OR = 1.99), *TRIM49* (Tripartite Motif Containing 49)*/NAALAD2* (N-acetylated alpha-linked acidic dipeptidase-2) (OR = 1.65) and *MYO16* (Myosin-16) (OR = 3.91) [43]. Another GWAS in a multiancestral cohort of children and adults with SLE (71/940 with osteonecrosis) identified an intronic variant in *WIPF1* (WAS/WASL interacting protein family member 1) to be associated with increased osteonecrosis risk that remained after accounting for corticosteroid exposure [44]. Studies highlight the need for structured investigation of the genetic contribution to specific manifestations and promise a deeper understanding of genetic factors shaping clinical heterogeneity in SLE.

**Table 1:** jSLE-associated common SNPs in genes also associated with “monogenic SLE”

Gene context	rsid	Chr	Pos (hg19)	AlleleA	AlleleB	Variant annotation	OR (95% CI)	*P*-value	References
*BANK1*	rs4637409	4q24	102 753,408 (hg19)	C		intronic	0.84 (0.80–0.87)	1.45 × 10^−17^	[17]
*BLK*	rs2061831	8p23	11 339,882 (hg19)	G		regulatory region	1.31 (1.25–1.37)	1.46 × 10^−40^	[17]
*IFIH1*	rs1990760	2	163 124,051 (hg19)	C	T	missense	1.11 (1.07, 1.11)	1.8210^−7^	[149]
	rs11891191	2	162 267,194 (hg38)	C	T	3’UTR variant (^∗^9C>T)	N/A	Disease-onset: 0.006; Activity: 0.005 (Female 0.01; non-European 0.01); Mucocutaneous: 0.003 (Female 0.008; non-European 0.01)^∗^	[42]
	rs1990760	2	162 267,541 (hg38)	C	T	missense	In linkage disequilibrium with rs3747517	Disease-onset: 2 × 10^–12^; Activity: 0.006 (Male 0.001); Mucocutaneous: 0.008 (Female 0.006)^∗^	
	rs3747517	2	162 272,314 (hg38)	C	T	missense	In linkage disequilibrium with rs1990760	Disease-onset: 2 × 10^−15^	
*IRAK1*	rs1059702	X	154 018,741 (hg38)	A	G	missense	N/A	Disease-onset: 0.006; Age: 0.002 (non-European 0.0002)^∗^	[42]
*RNASEH2C*	rs1308020	11	65 497,558 (hg19)	G	A	intergenic	0.87 (0.84, 0.87)	1.28 × 10^–9^	[150]
*TNFAIP3*	rs2230926	6	138 196,066 (hg19)	T	G	missense	1.88 (1.74, 1.88)	7.34 × 10^−59^	[151]
*TNIP1*	rs10036748	5	150 458,146 (hg19)	C	T	intronic	1.28 (1.23, 1.28)	5.65 × 10^−33^	[17,151]
*TLR7*	rs4830478	X	12 900,169 (hg19)	A		regulatory region	1.136 (1.09–1.18)	2 × 10^–9^	[18]
	rs3853839	X	12 887,911 (hg38)	C	G	3’UTR variant (^∗^881C>G)	N/A	Disease-onset: <10^–15^; Mucocutaneous: 0.004 (Female 0.01; European 0.03)^∗^	[42]

CI, 95% confidence interval; N/A, not available; OR, odds ratio. ^∗^ Spearmen's rho based on targeted panel-sequencing approach [14^▪^].

The combined consideration of risk alleles in polygenic risk scores (PRS) can increase predictive ability for disease expression and associated phenotypes [24,25^▪▪^]. Their accuracy and predictive value depend on the degree of genetic divergence between discovery and validation cohorts [26,45]. Early studies demonstrated differences in SLE-PRS distributions by ancestry, with people of Amerindian, South-Asian, East-Asian and African heritage exhibiting higher SLE-PRS when compared to Europeans [19]. Studies of SLE-PRS derived from large European cohorts demonstrated area under the receiver operating characteristic (ROC) curves (AUC) of 0.72 (95% CI: 0.69–0.74) [37^▪▪^]. Similarly, when PRSs are trained and tested in Chinese cohorts, SLE-PRS AUC reach 0.76 (95% CI: 0.74–0.78) [25^▪▪^]. As expected, the performance of European-derived SLE-PRSs in Chinese cohorts were modest (AUC: 0.62; 95% CI: 0.60–0.64) [25^▪▪^], emphasizing the importance of ancestral matching between discovery and test populations.

## RARE VARIANTS

The importance of rare high effect-size variants was first reported in the 1970s in the context of the complement system [46]. Although “monogenic SLE” is rare in human populations, variants commonly affect the same genes and pathways that exhibit common SLE-associated variants (Table 1) [3,11,12,14^▪^,^47^]. Through their high effect-size, rare variants shaped the current understanding of SLE pathogenesis [3] and facilitated the recognition that excessive IFN production plays a key role in SLE [3,48,49]. Increased accessibility allowed single centers and consortia to execute studies using whole exome or whole genome sequencing. As a result, rare variants have been reported in 3.5–15% of jSLE patients in different cohorts worldwide [13,14^▪^,^50^]. The true prevalence of “monogenic SLE” across age-groups and ancestries, however, cannot be estimated accurately.

Although we are just beginning to fully understand the genetic architecture of SLE, some key pathways have emerged (Table 2):

**Table 2:** Genes associated with “monogenic SLE”

Gene symbol	Gene name	Gene function	Functional impact	Reference
*ACP5*	Acid Phosphatase 5, Tartrate Resistant	Acid phosphatase	LoF	[152]
*ADAR1*	Adenosine Deaminase RNA Specific	RNA processing	LoF	[153]
*ADA2*	Adenosine Deaminase 2	Degrades extracellular adenosine	LoF	[154]
*BANK*	B Cell Scaffold Protein With Ankyrin Repeats 1	B cell receptor signaling	LoF	[155]
*BLK*	BLK Proto-oncogene, Src Family Tyrosine Kinase	B cell receptor signaling, B cell development	LoF	[155]
*C1QA*	Complement C1QA	Complement	LoF	[156,157]
*C1QB*	Complement C1QB	Complement	LoF	[158]
*C1QC*	Complement C1QC	Complement	LoF	[158]
*C1R*	Complement C1r	Complement	LoF	[159]
*C1S*	Complement C1Qs	Complement	LoF	[160]
*C2*	Complement C2	Complement	LoF	[54]
*C4A*	Complement C4A	Complement	LoF	[161,162]
*C4B*	Complement C4B	Complement	LoF	[161,163]
*CYBB*	Cytochrome b-245 β chain	Oxidase in phagocytes	LoF	[164]
*DDX58*	DEAD Box Polypeptide 58 (RIG-I)	dsRNA sensor	GoF	[70,165,166]
*DNASE1*	Deoxyribonuclease 1	DNA clearance	LoF	[167]
*DNASE1L3*	Deoxyribonuclease 1 like 3	DNA clearance	LoF	[61]
*DNASEII*	Deoxyribonuclease 2	DNA clearance	LoF	[58]
*FAS (TNFRSF6)*	Fas cell surface death receptor	Apoptosis	LoF	[168,169]
*FASL*	Fas Ligand	Apoptosis	LoF	[170]
*IFIH1(MDA5)*	Interferon Induced with helicase C	RNA sensor	GoF	[165,171]
*ISG15*	ISG15 ubiquitin-like modifier	Negative regulation of Type I IFN	LoF	[172]
*KRAS*	KRAS Proto-oncogene, GTPase	RAS/MAPK signaling	LoF	[173]
*LYN*	LYN Proto-oncogene, Src family tyrosine kinase	Regulates inhibitory signaling in B cells and myeloid cells	LoF	[174]
*MAN2B1*	Mannosidase αclass 2B member 1	Carbohydrate metabolism	LoF	[90]
*NEIL3*	Nei Like DNA Glycosylase 3	Glycosylase which initiates base excision repair	LoF	[175]
*PEPD*	Peptidase D	Collagen degradation	LoF	[176]
*PRKCD*	Protein kinase C δ	Serine/threonine kinase	LoF	[76,177]
*PSMA3*	Proteasome 20S Subunit α3	Proteasome	LoF	[178]
*PSMB4*	Proteasome 20S Subunit β4	Proteasome	LoF	[178]
*PSMB8*	Proteasome 20S Subunit β8	Proteasome	LoF	[178]
*PSMB9*	Proteasome 20S Subunit β9	Proteasome	LoF	[178]
*PSMB10*	Proteasome 20S Subunit β10	Proteasome	LoF	[178]
*PTPN2*	Protein tyrosine phosphatase, nonreceptor type 2	Negative regulator of JAK/STAT	LoF	[81]
*PTPN11*	Protein tyrosine phosphatase, nonreceptor type 11	RAS/MAPK signaling	LoF	[179]
*RELA*	RELA Proto-Oncogene, NF-KB subunit	NF-KB signaling	LoF	[180]
*RAG2*	Recombination activating 2	V(D)J Recombination	LoF	[181]
*RNASEH2A*	Ribonuclease H2 Subunit A	RNA processing	LoF	[153]
*RNASEH2B*	Ribonuclease H2 Subunit B	RNA processing	LoF	[153]
*RNASEH2C*	Ribonuclease H2 Subunit C	RNA processing	LoF	[158]
*SAT1*	Spermidine/Spermine N1-Acetyltransferase 1	Polyamine metabolism	LoF	[88]
*SAMHD1*	SAM and HD domain containing deoxynucleoside triphosphate triphosphohydrolase 1	Deoxyribonucleoside triphosphatase, inhibits reverse transcription	LoF	[66]
*SHOC2*	SHOC2, leucine rich repeat scaffold protein	RAS/MAPK signaling	LoF	[182]
*SLC7A7*	Solute carrier family 7 member 7	Cationic amino acid transporter	LoF	[183–186]
*SOCS1*	Suppressor Of Cytokine Signaling 1	Negative regulator of JAK/STAT	LoF	[82]
*TLR7*	Toll-like receptor 7	TLR signaling	GoF	[74^▪▪^,^75^,^76^]
*TMEM173 (STING1)*	Transmembrane protein 173	Detects cytosolic nucleic acids	GoF	[89]
*TREX1*	Three prime repair exonuclease 1	DNA clearance	LoF	[90]
*TNFAIP3*	TNFα Induced Protein 3	NF-KB signaling	LoF	[91]
*TNIP1*	TNFAIP3 Interacting Protein 1	NF-KB signaling	LoF	[92,187]
*UNC93B1*	Unc-93 Homolog B1	TLR signaling	LoF	[77]

GOF, gain-of-function; LOF, loss-of-function.

*Genetic variants in the complement pathway* remain some of the most prevalent variants reported in SLE [13,14^▪^,^46^,^51^]. Early-onset SLE or lupus-like phenotypes have been associated with the deficiency of complement factors, including C1q, C1r, C1s, C2, C3, C4A, and C4B [51–55]. Complement regulates important immune processes, and the loss of clearing apoptotic debris contributes to autoantibody formation and IFN expression [3,49].

*Defects in nucleic acid sensing and processing*, either by abnormal clearance or enhanced sensing can trigger IFN expression, inflammation and autoimmunity [3,49]. SLE-like phenotypes were reported in patients with variants in Deoxyribonuclease (DNAse) enzymes that play an important role in innate antiviral responses. Deficiency of DNAse-1 (*DNASE1*), a cytoplasmic endonuclease degrading dsDNA [56], and lysosomal DNAse-2 (*DNASE2*) deficiency were reported in early-onset SLE patients who may exhibit “atypical” clinical features, such as neonatal pancytopenia, polyarthritis and cholestasis [57^▪▪^,^58^]. Loss-of-function variants affecting the 3’-5’ exonuclease *TREX1* (*DNAse3*) were reported in familial chilblain lupus, early-onset SLE, and in Aicardi-Goutières syndrome (AGS) [48,59]. Recently, transmission disequilibrium testing confirmed that jSLE patients are enriched in rare variants, and identified rare variants in a diverse jSLE cohort, including loss-of-function in *HNRNPUL2*, encoding for the Heterogeneous Nuclear Ribonucleoprotein U Like 2, that plays a key role in double-stranded DNA repair [60^▪^]. Lastly, loss-of-function variants in *DNASE1L3*, an extracellular serum DNAse, were reported in six families [61]. DNAse-1L3 is responsible for extracellular dsDNA clearance. Its loss results in extracellular accumulation of immunogenic DNA (e.g. from neutrophil NETs), their recognition by innate toll-like receptors (TLRs) and IFN production that amplifies damage and extracellular DNA accumulation [62].

Disruption of RNA sensing has been linked with SLE. Variants in *RNASEH2A*, *RNASEH2B*, and *RNASEH2C*, encoding for the heterotrimeric RNAseH2 ribonuclease complex, were reported in SLE and AGS [63]. RNAseH2 processes RNA/DNA hybrids, a complex found during endogenous retrovirus transposition, in R-loops, and long interspersed nuclear elements (LINE-1) that trigger autoimmunity [64,65]. The innate antiviral SAMHD1 protein recognizes single-stranded (ss)RNA, ssDNA and self-dsDNA. Monogenic SLE, AGS, and increased IFN expression were reported in patients with *SAMDH1* loss-of-function variants [14^▪^,^66^–^68^]. The retinoic-acid-inducible-gene-I/RIG-I (*DDX58*) encodes for a cytosolic RNA sensor that activates mitochondrial antiviral signaling protein (MAVS) to induce IFNs and other inflammatory cytokines. Gain-of-function variants in *DDX58* were reported in Singleton-Merton syndrome [69], SLE and LN [70].

*Innate endosomal toll-like receptors (TLRs 3,7,8,9)* are important IFN activators [71]. Especially the importance of the lysosomal ssRNA sensor TLR-7 has been highlighted in SLE [72,73]. Recently, a *de novo TLR7* gain-of-function variant was discovered in a jSLE patient, enhancing TLR-7 activation through guanosine and cGMP [74^▪▪^]. This was validated by additional reports of gain-of-function variants in *TLR7* in additional patients, highlighting neuroinflammation as an important clinical feature of this genotype [75,76]. The critical role of TLR-7 in SLE was further demonstrated by missense variants in *UNC93B1*, encoding for a chaperone protein that binds TLRs in the endoplasmic reticulum, guides them to endo-lysosomes, and modulates TLR-7 responses to self-nucleic acid [77]. Loss-of-function impairs TLR-7 regulation, resulting in pro-inflammatory cytokine (including IFN) expression and systemic autoimmunity [77,78^▪▪^,^79^,^80^]. As TLRs are partially redundant and/or complementary, it appears likely that SLE-associated variants will also be detected in other endosomal TLRs.

*Defective regulation of JAK/STAT signaling* in jSLE has recently been highlighted by the identification of 6 “novel” loss-of-function variants in the Tyrosine-protein phosphatase nonreceptor type 2 encoding gene *PTPN2*[81]. PTPN2 regulates the JAK/STAT pathway through dephosphorylation of JAK and STAT proteins to prevent prolonged activity. SLE-associated variants contribute to elevated IL-2, type I and II IFN levels, immunodeficiency and autoimmunity [81]. These observations underscore the importance of negative regulation of JAK/STAT signaling to retain immune balance that was previously highlighted by early-onset autoimmunity associated with suppressor of cytokine signaling-(SOCS)1 haploinsufficiency [82].

*Dysregulated NF-κB signaling* was recently linked with SLE phenotypes. Heterozygous variants in *RELA* were reported in patients with early-onset SLE, where modified RelA/p65 proteins spontaneously localize to the nucleus, leading to a lower activation threshold of the NF-κB and elevated IFN expression [83]. Patients with loss-of-function variants in *TNFAIP3* encoding for the A20 chaperone protein in the NF-κB pathway demonstrate the connection of this pathway to autoimmunity. Loss of A20 function (also referred to as haploinsufficiency A20/HA20) associates with clinically variable phenotypes, including Behçet's disease, SLE, and JIA [14^▪^,^84^].

Abnormalities in immune cell signaling and metabolism – a variant in the serine/threonine protein kinase Cδ (*PRKCD*) was reported in three siblings with SLE [85], and subsequently validated in another, consanguineous, family [86]. Recently, siblings with jSLE and immunodeficiency were reported to have compound heterozygous variants in *PRKCD*[87^▪^]. The reduction in PKCδ activity leads to deficient B cell apoptosis and increased B cell proliferation [85]. A recent study described two families with X-linked recessive loss-of-function variants in *SAT1*[88], encoding for the Spermidine/spermine N1-acetyltransferase 1 (SSAT1) enzyme. SSAT1 regulates polyamines catabolism and is most highly expressed in neutrophils. Polyamines bind DNA and change its conformation from *B*-DNA to *Z*-DNA, which may trigger autoimmunity. Neutrophils from mice carrying SAT1 loss-of-function variants are more likely to produce neutrophil NETs. This adds to the body of previous studies reporting rare variants in genes coding for metabolic proteins such as *PEPD* (encoding for prolidase) and *MAND2B1* (mannosidase-alpha class-2B member-1) in patients with jSLE [89,90].

*Somatic, nongermline variants* may affect individual cell-lines and can be missed in currently used sequencing approaches. In SLE, reports on resulting mosaicism are mostly limited to aneuploidy syndromes that also increase the risk SLE development, including Klinefelter (47XXY) [91,92] and Triple X (47XXX) syndromes [93]. Recently, a patient with jSLE-like disease and mosaic tetrasomy affecting 9p24.3q12 (including the IFN cluster) was reported, which may contribute to increased IFN expression [94].

Moreover, heterozygous somatic mutations have been reported in the *NRAS* proto-oncogene (c.38A>G/p.G13C) of 4 jSLE patients that associate with reduced interactions of the GTPase NRAS with BCL-2 (B-cell lymphoma 2). Subsequently reduced apoptosis likely contributes to SLE-like disease with a lymphoproliferative phenotype [95], resembling clinical phenotypes observed in patients with Autoimmune Lymphoproliferative syndrome (ALPS) [96,97].

Mosaics affecting immune cell subsets have previously been reported in monogenic autoinflammatory diseases, namely cryopyrin-associated periodic syndrome (CAPS) [98–101], where mosaicism can result in untypically late disease-onset through clonal expansion [102]. Thus, somatic variants affecting abovementioned genes may also contribute to SLE and should move into the focus of future research.

Phenotypic variability in “monogenic SLE” has been reported. Results from WES/WGS in SLE cohorts suggests interplay between rare and common variants that alter disease phenotypes. The UK Biobank provides WES data from 454 787 participants (95% European) [103]. A recent study focusing on immune-mediated diseases including SLE suggested contributions from both common and rare variants to diseases, as well as pleiotropy across immune-mediated diseases [104^▪^]. While this highlights the functional convergence of common and rare variant associations with SLE, it does not necessarily explain disease discordance in genetically identical monozygotic twins [105]. Thus, additional acquired (namely epigenetic) events may be involved [15^▪^,^105^].

## EPIGENETIC CONTRIBUTION

Epigenetic mechanisms control gene expression through re-arrangement of nucleosomes and variable chromatin accessibility in a tissue-specific manner [7,8,16,106]. The addition of a methyl-group to the 5’ carbon position within cytidine-guanosine dinucleotides reduces recruitment of the transcriptional complex to regulatory regions [16,107]. Posttranslational histone modifications result in “opening” or “closure” of chromatin through changing electric charge [107,108]. Noncoding (nc)RNAs include long ncRNAs, that modulate chromatin conformation and control interactions between core promoters and distal regulatory elements [109], and micro-(mi)RNAs [107] that control the stability and integrity of mRNA [110]. Epigenetic marks change during cell and tissue differentiation, are heritable but reversible. They are susceptible to hormonal impact (e.g., estrogens) and environmental exposure (e.g. to infections, diet, medications) [7,106]. Thus, epigenetic marks are interesting candidates in the search for disease biomarkers and therapeutic targets across age-groups [3,111].

*DNA methylation*, the most studied epigenetic mechanism, contributes to variable genotype:phenotype correlations in inflammatory disease, including SLE [7,15^▪^,^106^,^112^]. DNA methylation profiles in immune cells have been linked with altered receptor/ligand interactions, transcription factor signaling and cytokine expression. Hypomethylation of the *CD70* promoter in CD4^+^ T cells from aSLE patients results in increased co-stimulatory CD70 surface expression and B cell stimulation [8,113–116] which was, however, not confirmed in PBMCs (Peripheral Blood Mononuclear Cells) from jSLE patients [115].

The lymphocyte-specific Lck tyrosine kinase is essential for the activation and function of naïve and effector T cells [117]. Hypomethylation of the *LCK* gene in CD4^+^T cells may therefore contribute to pathological T cell activation in jSLE [118,119]. The SLE-associated effector T cell phenotype may be favored further through inhibition of the transcription factor FOXP3 (Forkhead Box P3) that is essential for the development and function of regulatory T cells (Treg) [120–128]. Increased *FOXP3* methylation in jSLE likely impacts on its expression [129]. The signal transducer and activator of transcription (STAT) transcription factor family is controlled by balanced activity of Janus kinases (JAK) and suppressor of cytokine signaling (SOCS) family members. STAT transcription factors play a key role in multiple aspects of immune responses and effector T cell biology [130]. In PBMCs from jSLE patients, hypomethylation of the *JAK2* and hypermethylation of the *SOCS3* promoters contribute to imbalanced gene expression, increased IFN expression and immune activation [131]. Furthermore, epigenetic dysregulation of IFN-associated genes has been linked with their increased expression in PBMCs, CD4^+^/CD8^+^ T cells, CD19^+^ B cells, and CD16^+^ neutrophils from SLE patients [15^▪^,^108^,^113^,^118^,^132^]. Notably, several Differentially Methylated Positions (DMPs) between jSLE patients and controls are shared between immune cell subsets and affect IFN signaling [118]. One of these shared genes, *IFI44L*, is hypomethylated in adult [133–135] and pediatric patients [136]. Oligoadenylate synthetases (OAS)1 and 2 are IFN-induced genes involved in the innate antiviral response [137,138]. Hypomethylation of *OAS1* and *OAS2* and upregulated gene expression in CD8^+^ T cells and neutrophils associate with jSLE [118]. Furthermore, IFN dysregulation has been linked with LINE-1 that comprises 17–21% of the human genome and is involved in genome (in-)stability and evolution [139,140]. Hypomethylation of *LINE1* associates with IFN-regulated gene expression in autoimmune diseases, including SLE [140] where it associates with disease activity (in jSLE) [113].

Recently, phenotypic variation (severity of neurodevelopmental involvement) among AGS patients sharing the same genotype (*RNASEH2B* p.A177T) was linked with distinct DNA methylation profiles in PBMCs. Notably, DMPs associated with genes involved in IFN signaling associated with disease severity [15^▪^]. Thus, IFN-associated DNA methylation profiles may allow the prediction of disease activity and future patient stratification towards individualized treatment and care in patients with rare genetic forms of SLE/SLE-like disease and “classical” SLE.

*Posttranslational histone modifications* and the associated “histone code” are even more complex and incompletely understood, especially in children. However, genes affected by histone dysregulation in SLE (at least partially) overlap with pathologically methylated genes [3,106].

The IFN-induced transcriptional regulator interferon regulatory factor 5 (IRF5) plays a role in amplifying inflammation through pro-inflammatory cytokine expression [141]. Its transcription is controlled by histone acetylation, and the histone deacetylase inhibitor Trichostatin A reduces its expression, promising therapeutic considerations [40].

**FIGURE 1:**
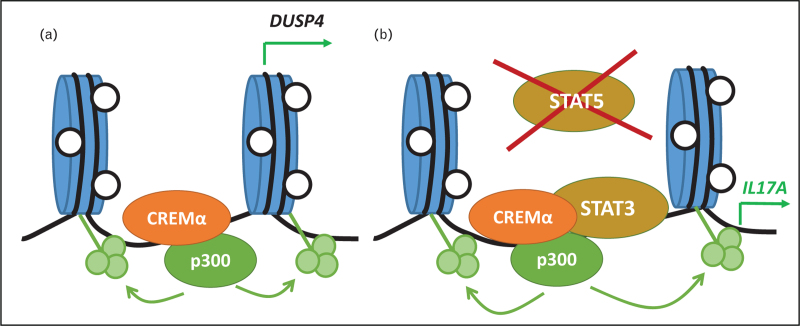
The transcription factor CREMα instructs epigenetic remodeling in T cells from SLE patients. (a) CREMα recruits to the *DUSP4* proximal promoter where it instructs histone acetylation (green circles) through co-recruitment of the histone acetyltransferase p300. Resulting epigenetic opening allows for the expression of the protein dual specificity protein phosphatase (DUSP)4. (b) At the same time, CREMα recruits to regulatory elements along the *IL17* cluster where it instructs DNA demethylation (open circles) and histone acetylation (green circles) through p300. Increased expression of DUSP4 (from a) results in an imbalance between phosphorylated STAT3 and STAT5, favoring STAT3 signaling. This results in increased recruitment of STAT3 alongside CREMα, both trans-activators of IL-17 transcription.

STAT5 controls the balance between Treg and effector Th17 cells [142]. In CD4^+^ T cells, the transcription factor cAMP Responsive Element Modulator (CREM)α recruits to the *Dual Specificity Protein Phosphatase (DUSP)4* promoter, which results in co-recruitment of the histone acetyltransferase p300, epigenetic “opening” and increased DUSP-4 expression [128,143]. This results in DUSP-4-mediated STAT5 de-phosphorylation and imbalanced STAT3/STAT5 signaling, favoring IL-17A and reducing IL-2 expression, hallmarks of SLE T cells [128,144] (Fig. 1). Because CREMα is induced by estrogen receptor signaling, this mechanism may contribute to the female predominance and phenotypical differences between pre and postpubertal SLE patients [5,145].

Lastly, in addition to functional impairment of enzymatic activity, mutations in *RNASEH2B* affect its interaction with the chromatin silencing complex (CoREST) that, among others, comprises HDAC2 and Lysine-Specific Histone Demethylase (KDM)1A. This suggests coordination between histone modifications and the degradation of RNA:DNA hybrids and its dysregulation in SLE [146].

*Dysregulated expression of miRNAs* is associated with DNA methylation and histone modifications [147,148]. Recent studies highlight the involvement of altered miRNA expression in the dysregulation of innate and damage-associated pathways, effector and regulatory T cell function in jSLE. Associations with organ involvement and/or disease activity and damage promise potential as future markers of disease activity and progression (Table 3).

**Table 3:** Micro-RNAs associated with jSLE and other systemic autoimmune/inflammatory diseases (may be incomplete)

miRNA							
Up-regulated	Down-regulated	Cell type/tissue	mRNA target(s)	Function(s)	Proposed biomarker potential	jSLE context	Additional inflammatory diseases context
	MiRNA-155	PBMCs	PP2A_C_ and IL-2	Forced expression of miRNA-155 decreases relative expression of PP2Ac and increases IL-2 release in c PBMCs *in vitro*. Ex vivo expression of PP2AC mRNA correlates with SLEDAI scores. Patients with SLE exhibit increased activity of the PP2A catalytic subunit resulting in decreased IL-2 production in T cells [188].	MiRNA-155 inversely correlates with SLEDAI scores, proteinuria and correlated with total leukocyte counts.	[189]	RA [190], LSc [191], SjS [192], MS [193], JIA [194]
MiRNA-146a		Plasma (circulating exosomal miRNA)	TNF Receptor Associated Factor 6 (TRAF6)	Circulating exosomal miRNA-146a may inhibit lupus-induced inflammation through down-regulation of TRAF6 and regulate IFN production.	MiRNA-146a inversely correlates with SLEDAI-2K, antidouble stranded DNA and urine protein creatine.	[195]	RA, SLE, IBD, MS, psoriasis, GD, GO, SjS, T1D, AS, SSc, DM, BD, DM [196]
	MiRNA-125a	Plasma	Unknown	MiRNA-125a expression negatively associated with IL-17 levels, which may contribute to Th17 phenotype in SLE.	MiRNA-125a inversely correlates with SLEDAI-2K, SLICC scores, ESR and proteinuria.	[197]	RA [198], SLE [199], GD [200], HD [200]
	MiRNA-145	Renal vascular cells	Unknown	The expression of miRNA-145 is linked to the degree of renal vascular lesions (RVL) in lupus nephritis (LN). MiRNA-145 is negatively regulated by PDGF-BB. PDGF-BB-induced proliferation and migration of human vascular smooth muscle cells (HVSMC) is inhibited by miRNA-145. MiRNA-145 may promote apoptosis of HVSMCs and play a role in vascular injury.	MiRNA-145 may allow noninvasive monitoring of vascular lesions (negative correlation) in juvenile lupus nephritis patients.	[201]	SLE [202], MS [203], SSc [204], CD [205]
	MiRNA-9–5p	PBMCs	NFKB1	MiRNA-9-5p and miRNA-125b-5p are downregulation in jSLE patients when compared to controls. NFKB1 expression is downregulated and TRAF6 is upregulated in jSLE patients when compared to the healthy controls.	Both miRNAs may be markers of the disease, but no differences were observed between active and inactive jSLE.	[206]	MS [207], T1D [208], SLE [209]
	MiRNA-125b-5p		TRAF6			[207]	Psoriasis [210], SLE [211], RA [212]
	MiRNA-200a	Serum	Unknown	Serum levels of miR-200a are lower in jSLE patients as compared to healthy participants.	Disease activity: MiRNA-200a negatively correlates with SLEDAI (r = -0.425), ESR (r = -0.284), CRP (r = -0.338), BUN (r = -0.263) and Scr (r = -0.345), while it positively correlates with C3 (r = 0.631), C4 (r = 0.524) and ALB (r = 0.394). Diagnostic for SLE: AUC 0.8379 (cut-off value = 2.225, sensitivity = 70%, specificity = 70%). Diagnostic for LN: AUC 0.7619 (cut-off value = 2.005, sensitivity = 80%, specificity = 76%)	[213]	MS [214]

ALB, albumin; AS, ankylosing spondylitis; BD, Behçet's disease, BUN, blood urea nitrogen; CD, Crohn disease; CRP, C-reactive protein; DM, dermatomyositis; ESR, erythrocyte sedimentation rate; GD, Graves ’ disease; GO, Graves’ ophthalmopathy (orbidopathy); HD, Hashimoto disease; IBD, inflammatory bowel disease; JIA, juvenile idiopathic arthritis; LN, lupus nephritis; LSc, localised scleroderma; MS, multiple sclerosis; MS, multiple sclerosis; PDGF-BB, platelet derived growth factor BB; RA, rheumatoid arthritis; scr, serum creatinine; SjS, primary Sjogren syndrome; SLE, systemic lupus erythematosus; SLEDAI, systemic lupus erythematosus disease activity index.SLEDAI-2K, systemic lupus erythematosus disease activity index 2000; SLICC, 2012 Systemic Lupus Collaborating Clinics classification criteria for SLE; SSc, systemic sclerosis; T1D, type 1 diabetes mellitus.

## CONCLUSION

SLE is a patho-mechanistically complex autoimmune/inflammatory disease. Though most studies have been performed in aSLE cohorts of European ancestry, we are beginning to understand genetic and environmental impacts that shape phenotypic differences between age-groups and ancestries. Genetic predisposition is necessary to develop disease, and genetic burden defines the age at disease-onset. First attempts to predict organ involvement and disease outcomes promise potential for future patient stratification towards individualized treatment and care. While rare variants with high effect size only affect a minority of SLE patients across age-groups, they transformed the understanding of more common forms of SLE. Phenotypic variability among patients with “monogenic SLE” underscores a role for additional common variants and epigenetic variability. A better understanding of epigenetic marks will allow their inclusion in diagnostic, prognostic and therapeutic considerations.

## Acknowledgements

*L.T.H. holds a Tier 2 Canada Research Chair in Genetics of Rare Systemic Inflammatory Diseases, and research is supported by Childhood Arthritis and Rheumatology Research Alliance (CARRA), Lupus Research Alliance (LRA), U.S. Department of Defense, Lupus Foundation of America, Gary Hurvitz Centre for Brain & Mental Health Catalyst Grant. L.B.L. was funded by the NIAMS Intramural Program. C.M.H.'s research is supported by Versus Arthritis UK, LUPUS UK, Merck MISP, the Alder Hey Children's Kidney Fund, FAIR: Funding Autoimmune Research, Scleroderma and Raynaud's UK, the Alder Hey Children's Charity, the NIHR Great Ormond Street Biomedical Research Centre (NIHR GOSH BRC), the NIHR Alder Hey Clinical Research Facility (NIHR AH CRF).*

### Financial support and sponsorship

*None.*

### Conflicts of interest

*L.T.H. consults for J&J. C.M.H. received unrestricted research support from Novartis (psoriasis) and Merck (MISP programme, lupus nephritis).*

## REFERENCES AND RECOMMENDED READING

Papers of particular interest, published within the annual period of review, have been highlighted as:

▪ of special interest
▪▪ of outstanding interest
